# Transmission-blocking activity of antimalarials for *Plasmodium vivax* malaria in *Anopheles darlingi*

**DOI:** 10.1371/journal.pntd.0011425

**Published:** 2023-06-16

**Authors:** Alice O. Andrade, Najara A. C. Santos, Alessandra S. Bastos, José D. C. Pontual, Jéssica E. Araújo, Alexia M. V. Silva, Leandro N. Martinez, Alzemar A. Lima, Anna Caroline C. Aguiar, Carolina B. G. Teles, Jansen F. Medeiros, Dhelio B. Pereira, Joseph M. Vinetz, Ricardo T. Gazzinelli, Maisa S. Araújo

**Affiliations:** 1 Plataforma de Produção e Infecção de Vetores da Malária (PIVEM), Laboratório de Entomologia, Fiocruz Rondônia, Porto Velho, Rondônia, Brazil; 2 Programa de Pós-Graduação em Biologia Experimental, Fundação Universidade Federal de Rondônia, FIOCRUZ Rondônia, Porto Velho, Rondônia, Brazil; 3 Ambulatório de Malária, Centro de Pesquisa em Medicina Tropical, Porto Velho, Rondônia, Brazil; 4 Plataforma de Bioensaios de Malária e Leishmaniose da Fiocruz (PBML), Fiocruz Rondônia, Porto Velho, Rondônia, Brazil; 5 Universidade Federal de São Paulo, Departamento de Biociência, Santos, São Paulo, Brazil; 6 Section of Infectious Diseases, Department of Internal Medicine, Yale School of Medicine, New Haven, Connecticut, United States of America; 7 Laboratório de Imunopatologia, Instituto René Rachou, Fundação Oswaldo Cruz, Belo Horizonte, Minas Gerais, Brazil; 8 Department of Medicine, University of Massachusetts Medical School, Worcester, Massachusetts, United States of America; University of Buea, CAMEROON

## Abstract

Malaria is caused by parasite of the genus *Plasmodium* and is still one of the most important infectious diseases in the world. Several biological characteristics of *Plasmodium vivax* contribute to the resilience of this species, including early gametocyte production, both of which lead to efficient malaria transmission to mosquitoes. This study evaluated the impact of currently used drugs on the transmission of *P*. *vivax*. Participants received one of the following treatments for malaria: i) chloroquine [10 mg/kg on day 1 and 7.5 mg/kg on day 2 and 3] co-administered with Primaquine [0.5 mg/kg/day for 7 days]; ii) Chloroquine [10 mg/kg on day 1 and 7.5 mg/kg on day 2 and 3] co-administered with one-dose of Tafenoquine [300 mg on day 1]; and iii) Artesunate and Mefloquine [100 mg and 200 mg on day 1, 2 and 3] co-administered with Primaquine [0.5 mg/kg/day for 14 days]. Patient blood was collected before treatment and 4 h, 24 h, 48 h and 72 h after treatment. The blood was used to perform a direct membrane feeding assay (DMFA) using *Anopheles darlingi* mosquitoes. The results showed 100% inhibition of the mosquito infection after 4 h using ASMQ+PQ, after 24 h for the combination of CQ+PQ and 48 h using CQ+TQ. The density of gametocytes declined over time in all treatment groups, although the decline was more rapid in the ASMQ+PQ group. In conclusion, it was possible to demonstrate the transmission-blocking efficacy of the malaria vivax treatment and that ASMQ+PQ acts faster than the two other treatments.

## Author summary

Vivax malaria is the most prevalent and widespread human malaria outside the African continent. The control of vivax malaria is based on vector control and effective radical cure of infected people. However, the elimination of the sexual stages of *P. vivax* present in infected people is important to block malaria transmission from human to mosquito vector. Here, we assessed the potential of currently used antimalarial drugs for vivax malaria therapy for blocking malaria transmission in the mosquito vector *An. darlingi*. We observed that the antimalarials tested were able to block the infection of mosquitoes; however, treatment based on the artemisinin combination proved to be the fastest for blocking transmission in mosquitoes, using blood taken 4 h after treatment. As such, patients in endemic areas who receive the first line vivax malaria treatment, chloroquine and primaquine, could need protection from bites of the malaria vector for at least 24 h after the initial dose in order to interrupt malaria transmission. Our observations provide an overview of the transmission-blocking profile of antimalarials currently used in Brazil for the treatment of vivax malaria and they also provide a platform for the evaluation of new antimalarials, such as tafenoquine or other compounds for combating the sexual stages of *P*. *vivax*, which may impact the epidemiology of malaria.

## Introduction

Malaria is caused by a parasite of the genus, *Plasmodium*, which remains of global public health significance [1]. The malaria parasite has a complex life cycle in which *Plasmodium* sporozoites are injected by infected *Anopheles* mosquitoes into a mammalian host. They first localize to liver, where they replicate into thousands of merozoites that invade red blood cells. Some merozoites differentiate into gametocytes [2] and, within the mosquito midgut, male and female gametes fuse to form zygotes, which develop into the motile and invasion ookinete that penetrates the midgut wall to form an oocyst [3]. Over the next approximately 14 days, oocysts mature under the basal lamina of the midgut epithelial surface to form sporozoites that migrate to the mosquito salivary glands, where they remain ready to initiate a new mammalian infection when injected into a new human with the next blood feeding [4,5].

*Plasmodium vivax* is the most prevalent and widespread human malaria parasite outside Africa [1]. Several biological characteristics of *P*. *vivax* contribute to the resilience of this species, as compared to *P*. *falciparum*, including: (i) *P*. *vivax* has a lower minimum temperature requirement for development in the mosquito, thus facilitating distribution in temperate and tropical climates [6]; (ii) *P*. *vivax* gametocyte production begins earlier in infection and is continuous, which contributes to more efficient transmission to mosquitoes [7,8] and (iii) *P*. *vivax* parasitemia is constrained to low levels due to selective invasion of reticulocytes, which elicits rapid clinical immunity in the host so that a large proportion of infections are subclinical and subpatent [9].

These biological characteristics of *P*. *vivax* result in a continued challenge for malaria control [10]. One strategy to control vivax malaria is the implementation of an effective radical cure, [7] which radical cure aims to kill the dormant hypnozoite stages of *P*. *vivax* responsible for relapses weeks or months after the initial infection [11]. For more than 70 years, the first-line therapy for radical cure of vivax malaria has been primaquine. Recently, tafenoquine has been implemented and has proven to be a more effective treatment since the adherence to antimalarial chemotherapy is improved and relapses are thus avoided [12].

In cases of recurrence of vivax malaria, other antimalarials can be combined for a more effective treatment, for example, artemisinin-combination treatments (ACTs). In Brazil, the Health Ministry recommend, in cases of malaria vivax recurrence between 5 to 60 days after first-line treatment, a combination of artemether/lumefantrine or artesunate/mefloquine to target asexual stages, plus primaquine due to its hypnozoiticidal action [13].

Besides eliminating the asexual stages responsible for patient’s symptoms and avoiding relapses by eliminating the hypnozoites in the liver, an effective antimalarial treatment should prevent the human to mosquito to human transmission [14]. This is particularly important to prevent *P*. *falciparum* transmission, since the gametocyte can survive longer than asexual stages [14]. As *P*. *vivax* development forms gametocytes at the beginning of the infection, the human hosts are already capable of infecting the anopheline vectors before the diagnosis and administration of antimalarial drugs [15,16]. Fortunately, the *P*. *vivax* gametocytes are more sensitive to the current antimalarials, which kill asexual stages, than *P*. *falciparum* [17]. Therefore, an effective combination of antimalarials may reduce the transmissibility of *P*. *vivax* from the patient to mosquito vector after starting their treatment [15].

The transmission-blocking activity of antimalarial drugs or of potential compounds can be gametocytocidal, which act eliminating the gametocyte, or sporontocidal, which act against stages of the sporogonic cycle of *Plasmodium*. However, there are not many studies on gametocytocidal or sporontocidal activity, and because of the lack of reproducible continuous culture methods, there is no *in vitro* model for assessing gametocytocidal drug activity against *P*. *vivax* directly [18]. The efficacy of antimalarial drugs in blocking transmission, as well potential compounds against *P*. *vivax* has been studied in experiments with the anopheline mosquito vector [17,19,20].

In this study, we aimed to understand how the currently used drugs in vivax malaria case management impact the transmission of *P*. *vivax* in the main malaria vector of Amazon region, the *Anopheles darlingi*. This study was conducted to evaluate the first-line treatment for vivax malaria, chloroquine and primaquine, the combination of artesunate and mefloquine recommended for patients suffering from relapses or recrudescence and, additionally, after the studies for the implementation of tafenoquine started to be conducted in the study area [13,21], the combination chloroquine and tafenoquine.

## Methods

### Ethics statement

Informed written consent from patients was obtained before patient enrollment. This study was reviewed and approved by Ethics Committee of the Centro de Pesquisa em Medicina Tropical (CEPEM), under approval number: protocol #28176720.9.0000.0011.

### Study area

The study was carried out in the city of Porto Velho, Rondônia state, and the subjects were patients with a positive vivax malaria test result from the CEPEM Malaria Clinic between August 2021 and January 2022. Porto Velho is in a malaria-endemic area in the Amazon region of Brazil. It attends approximately 50 people a day and performs routine malaria microscopy.

Malaria transmission in Porto Velho occurs throughout the year, though there is generally an increase from February to June, after the peak of the rainy season, a decrease during the dry season (June to August) and an additional peak of malaria occurred in September to October, which is the beginning of the rainy season. The cases are mainly reported in the rural and peri-urban areas of Porto Velho, with cases in males predominating (65.67%). The most-affected age groups were 20–39 years (40.28%) and 40–59 years (31.28%), which together represent 71.56% of cases [22]. *Plasmodium vivax* and *An. darlingi* are the predominant *Plasmodium* species and vector of the malaria parasite, respectively [22–27]. Malaria transmission in Porto Velho is low with an incidence rate of 9.6 cases per 1000 inhabitants. However, some locations in Porto Velho have Malaria Annual Parasite Index (API) ranging from 30 to 2,950 with an average API of 1189.02, and is considered high risk [22].

Chloroquine with primaquine has always been the first-line treatment for uncomplicated malaria in Rondônia since the 1940; however, tafenoquine is now included in the treatment of vivax malaria in Porto Velho and Manaus, Amazonas [13,21,28]. In case of recurrence between 5 and 60 days, a new treatment regimen that is more effective is recommended, such as artemether/lumefantrine or artesunate/mefloquine with primaquine, as described by Goller et al. [29].

### Inclusion and exclusion criteria of volunteers

Patients presenting at the malaria clinic with suspected acute uncomplicated malaria were screened for eligibility. Inclusion criteria were age from 18 and 85 years, microscopy-confirmed *P*. *vivax* mono-infection, a parasitemia level of 500 or more parasites per μL, ability to swallow oral medication and willingness to abide by the study protocol and be available for the stipulated follow-up visits. Exclusion criteria were evidence of severe malaria or danger signs, known allergy to trial medicines, being pregnant, being a member of the indigenous population and residing outside the study area.

### Study design

**Fig 1:**
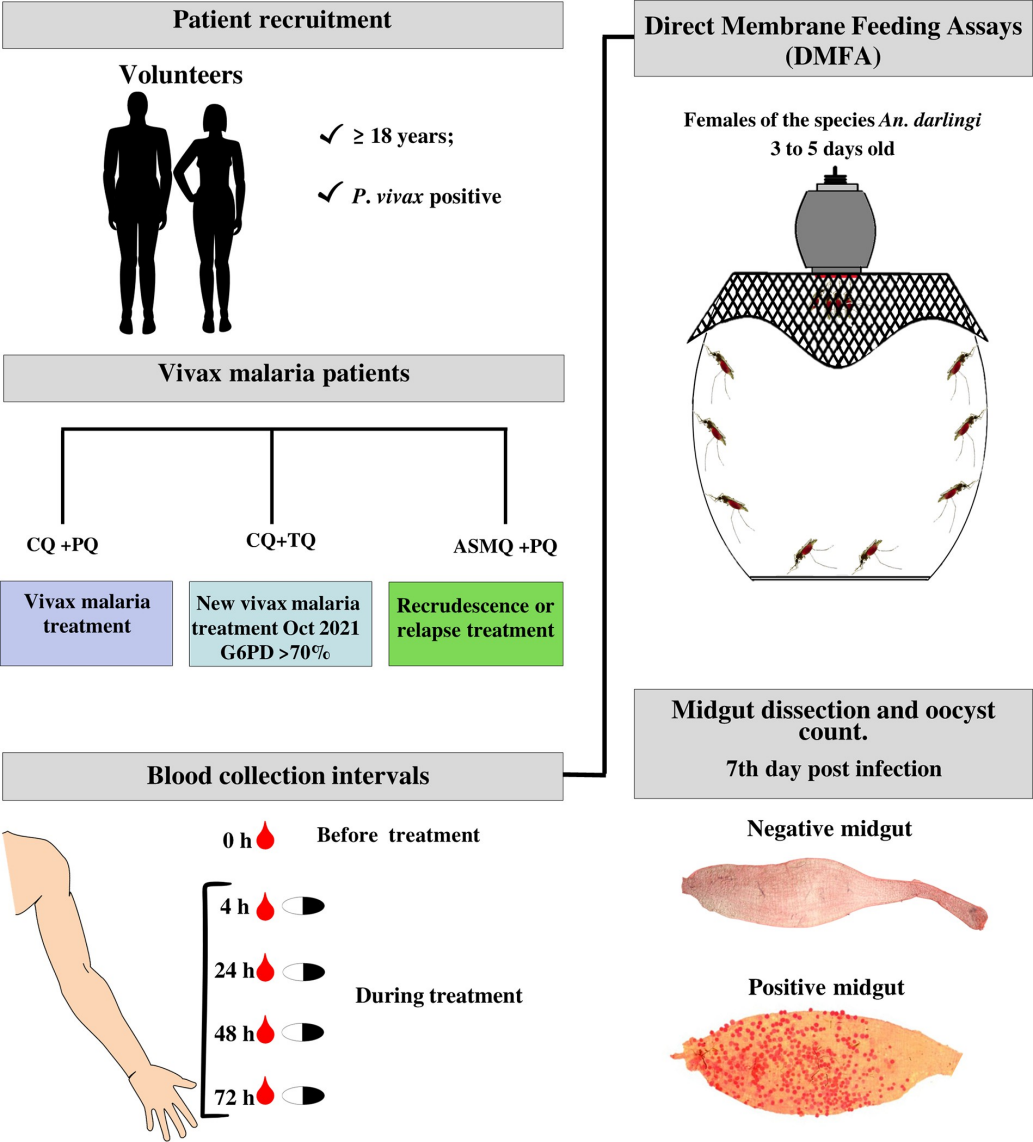
Schematic and summarized representation of our study design for assessment of transmission blocking activity of vivax malaria treatments.

Subjects were recruited at CEPEM. Direct membrane feeding assays were carried out at the Production and Infection of Malaria Vectors Platform—PIVEM/Fiocruz Rondonia. Subjects who received either one of these malaria treatments: i) Chloroquine [10 mg/kg on day 1 and 7.5 mg/kg on day 2 and 3] co-administered with Primaquine [0.5 mg/kg/day for 7 days]; ii) Chloroquine [10 mg/kg on day 1 and 7.5 mg/kg on day 2 and 3] co-administered with one-dose of Tafenoquine [300 mg on day 1]; and iii) Artesunate and Mefloquine [100 mg and 200 mg on day 1, 2 and 3] co-administered with Primaquine [0.5 mg/kg/day for 14 days], were identified as the following groups: CQ+PQ, CQ+TQ and ASMQ+PQ, respectively (Fig 1).

The CQ+PQ group were enrolled before the inclusion of tafenoquine as a vivax malaria treatment in Porto Velho, and the CQ+TQ group were enrolled from October 2021 onwards, when tafenoquine was included as a vivax treatment in Porto Velho, Rondonia state, and Manaus, Amazonas state, after a previous glucose-6-phosphate dehydrogenase (G6PD) test [13,28]. Patients that returned and still had vivax malaria after 14 to 60 days despite treatment with CQ+PQ were enrolled into the ASMQ+PQ group. In this study, recurrences were confirmed on days 14, 28 and 42 after initiating the treatment. Whether a recurrence occurred, the treatment followed the protocol described by Goller et al. [29], which is commonly used in Brazil [30].

All drugs were administered orally with food, and were provided without cost to all participants. The first blood collection (0 h), considered the control, was conducted at CEPEM before initiating the treatment, and the first dose of treatment was initiated immediately after blood collection (under supervision at the recruitment clinic). After initiation of treatment, all subsequent blood collections (4 h, 24 h, 48 h and 72 h) were carried out by a visiting health worker at the volunteers’ residence. The collections were carried out before administration of the drug and under full supervision (Fig 1).

Blood samples were collected in heparinized (9 mL) Vacutainer tubes, maintained in a water flask at 37°C and immediately transported to PIVEM, which took less than 40 minutes. At PIVEM, the heparinized blood was used for the direct membrane feeding assay (DMFA) and a thick blood smear was prepared in duplicate using 10 μL of whole blood for quantification of parasitemia and gametocytemia via microscopy.

### *Plasmodium vivax* peripherical parasitemia and gametocyte counts

Thick blood smears from each patient and from each timed blood collection were prepared then, Giemsa stained and examined under light microscopy (x100) with an oil immersion lens. The density of the gametocyte and asexual stages were estimated by counting parasites per 800 leukocytes according to WHO (2010) [31].

### Mosquito colony

The *An*. *darlingi* colony used in this study was established in 2018 by Araujo et al. [32]. The mosquitoes were maintained in the PIVEM insectary at 26 ± 1°C with a relative humidity of 70% ± 10%, and with a 12-hour light/dark cycle. Larvae were reared in 1 L of ddH_2_O, with a population density of 150 to 200 larvae per plastic pan and were fed every day with TetraMin Marine fish food. Pupae were collected and placed in cages (35 x 35 cm), and adult mosquitoes were provided with a 15% honey solution *ad libitum* and fed on rabbit blood to produce the next generation of mosquitoes.

### Membrane feeding and mosquito dissection

Three- to five-day-old *An*. *darlingi* mosquitoes, deprived of sucrose overnight, were placed in small plastic cups (80 mosquitoes per cage) and used for each assay. Two milliliters of heparinized blood from each timed blood collection (0 h, 4 h, 24 h, 48 h and 72 h) was added to a 5-cm-diameter glass membrane fitted with a parafilm membrane and maintained at 37°C during the whole experiment. After 30 min, unfed mosquitoes were removed and eliminated in 70% ethanol. Fully fed mosquitoes were kept under the same insectary conditions as described above. Cotton pads soaked in 15% honey solution were provide for subsequent days until the dissection of the mosquitoes. Equal numbers of mosquitoes (40 mosquitoes) were dissected on day 7 post blood feeding for assessing the oocyst load in the midgut. Each midgut was stained with 0.2% commercial mercurochrome and examined for presence of oocysts to determine the prevalence. The number of oocysts in each midgut were counted to determinate the infection intensity (number of oocysts per mosquito).

### Data analysis

Statistical analyses were carried out using GraphPad Prism (v.9). For all analyses, the threshold for statistical significance was set at p<0.05. Participants were considered infectious when at least one oocyst were found in mosquito’s midgut from each treatment group and time. The percentage of infectious patients in the timepoints after starting the treatment was compared with the control group (0 h) using Fisher’s exact test. Prevalence of infection in the mosquitoes at the different times after starting treatment was compared with the control (0 h) using a pairwise chi-squared test and Bonferroni correction for multiple comparisons. Similarly, comparisons of oocyst intensity, asexual parasitemia and gametocytemia were carried out using the Kruskal-Wallis test and Dunn’s multiple comparisons test. The inhibition of intensity and prevalence in each treatment at each timepoint of the study were also estimated. The inhibition parameters are the percentage reduction (relative to baseline) in the number of oocysts per mosquito and mosquito infection rate at the given timepoints. The inhibition prevalence was calculated as: 100 x [1 –(proportion of mosquitoes with oocyst in the test group)/(proportion of mosquitoes with oocyst in the control group)]; and the inhibition intensity: 100 x [1 –(mean number of oocysts in the test group)/(mean number of oocyst in the control group)]. Positive values denote a reduction in the percentage of mosquitoes infected and the number of oocysts per mosquitoes, negative values denote increases.

## Results

Twenty-four vivax malaria volunteers were enrolled in our study: CQ+PQ (n = 11), CQ+TQ (n = 7) and ASMQ+PQ (n = 6). However, 5 volunteers from the CQ+PQ group and 1 volunteer from the CQ+TQ group were excluded from study because they were not at home during a visit, or the assays could not be completed due to COVID-19-related constraints. Additionally, experiments which the mean oocyst account in the control group was less than 2.5 were excluded of analysis (S1 Table) due the impact of drug on parasites in these experimental groups may not be the real effect of the drugs [33].

Participant characteristics were similar between the treatment groups, and these are presented in Table 1.

**Table 1:** Baseline characteristics.

	CQ+PQ	CQ+TQ	ASMQ+PQ
	(n = 4)	(n = 4)	(n = 4)
Age (years)			
Mean (SD)	41.3 (22.9)	42.3 (14.4)	35.0 (9.1)
Range	21–64	24–59	23–45
Sex, n (%)			
Female	2 (50.0)	2 (50.0)	1 (25.0)
Male	2 (50.0)	2 (50.0)	3 (75.0)
Gametocyte density by microscopy (parasites/μL) (range)	600 (60–1680)	255 (0–510)	360 (30–930)
Asexual parasites density by microscopy (parasites/μL)(range)	18990 (3000–34110)	3135 (2130–16290)	9945 (1170–31680)
Patient infectious to mosquitoes n (%)	4 (100)	4 (100)	4 (100)
Percentage of mosquitoes infected (range)	95.0 (89.7–100)	95.6 (92.5–100)	83.8 (52.5–100)

Gametocyte and asexual density are represent in median. Parasitemias and infecious to mosquitoes data are before treatment.

**Fig 2:**
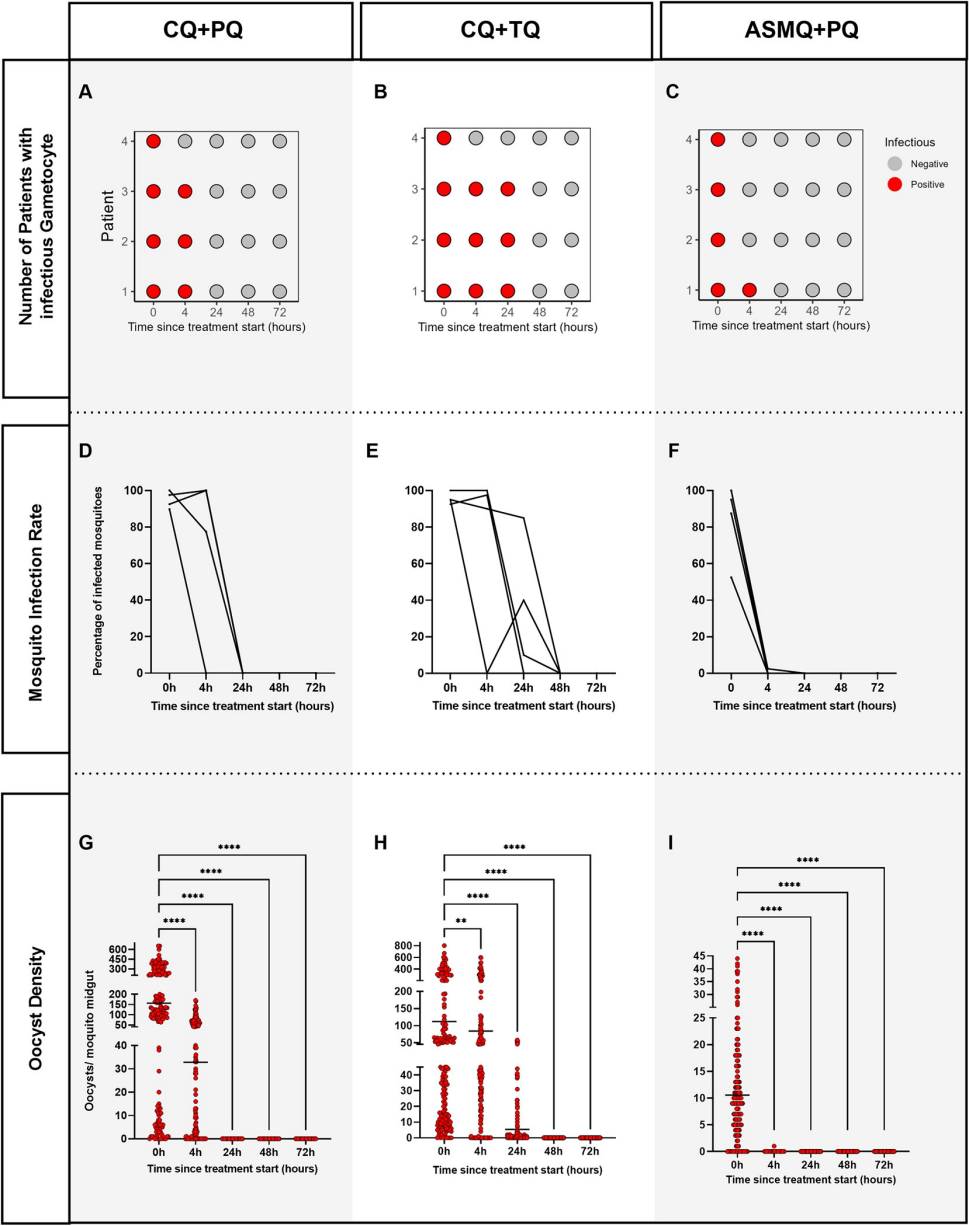
Panel shows the parameters for number of patients with infectious gametocyte, mosquito infection rate and oocyst density after the treatment with chloroquine and primaquine (CQ+PQ), chloroquine and tafenoquine (CQ+TQ) and artesunate/mefloquine and primaquine (ASMF+PQ) at 0 h before of treatment and at 4 h, 24 h, 48 h and 72 h after starting treatment. Graphs A, B, C show the number of patients with infectious gametocyte. Red color represents the time when patient was infected while gray color represents the time when the patient was uninfected. D, E, F show the mosquito infection rate via DMFA using blood samples. G, H, I show the oocyst density. Each circle represents a mosquito’s midgut. All dissected mosquitoes are shown. The lines of dots represent the mean. The Kruskal-Wallis test and Dunn’s multiple comparisons was used to compare groups. For all analyses, comparisons were made between the control group (0 h) and the timepoints after start treatment. Only significant differences are shown, ** p = 0.0018; **** p<0.0001.

The number of mosquitoes dissected per group varied between 39 to 40. Before treatment (0 h), all participants were infectious to mosquitoes (Fig 2A–2C and S1A Table).

**Fig 3:**
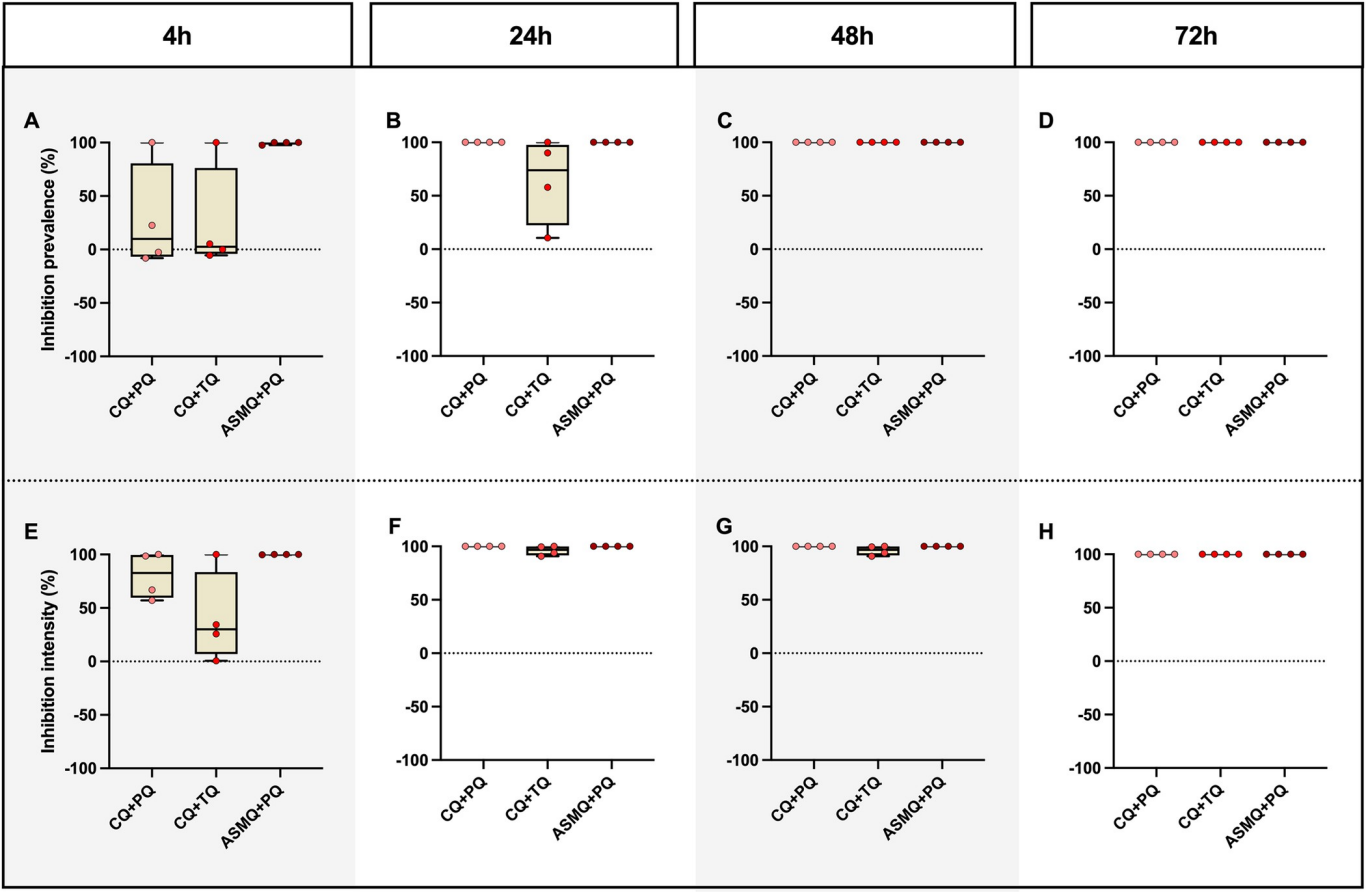
Assessment of vivax malaria treatment effects on oocysts of *Anopheles darlingi*, expressed as the prevalence inhibition and intensity inhibition. See S1 Table.

At 4 h after the start of treatment, three of the four participants from the CQ+PQ and CQ+TQ groups, and only one of the four participants in the ASMQ+PQ group were infected by *An*. *darlingi* (Fig 2A–2C and S2A Table). In the individuals who were infectious after the start of treatment, the mean of prevalence inhibition at 4 h after the start of treatment was 27.96% (range: -2.56 to 100) for individuals treated with CQ+PQ. For individuals treated with CQ+TQ, inhibition was 24.96% (range: -5.41 to 100) and, for individuals treated with ASMQ+PQ, inhibition was 99.38% (range 97.50 to 100); and the inhibition intensity was 80.63% (range: 57.11 to 100), 40.19% (range: 0.57 to 100) and 99.95% (range: 99.82 to 100) for CQ+PQ, CQ+TQ and ASMQ+PQ, respectively (Fig 3A and 3E and Tables 2 and S2). Statistical analysis showed that the prevalence of infected mosquitoes decreased significantly after initiating any of the three treatments (Fig 2D–2F and S2B Table). In the same way, the mean oocyst intensity was significantly different at 4 h after beginning of treatment compared with 0 h (time before treatment) for all treatments (Fig 2G–2I and S2B Table).

**Table 2:** Inhibition of Intensity and prevalence in mosquito infection for individuals who were infectious to mosquitoes after beginning of treatment.

		4h		24h		48h		72h	
	n	Inhibition of Intensity % (range)	Inhibition of Prevalence % (range)	Inhibition of Intensity % (range)	Inhibition of Prevalence % (range)	Inhibition of Intensity % (range)	Inhibition of Prevalence % (range)	Inhibition of Intensity % (range)	Inhibition of Prevalence % (range)
CQ+PQ	4	80.63	27.96	100.0	100.0	100.0	100.0	100.0	100.0
		(57.11 to 100)	(-2.56 to 100)	(100 to 100)	(100 to 100)	(100 to 100)	(100 to 100)	(100 to 100)	(100 to 100)
CQ+TQ	4	40.19	24.96	96.10	66.84	100.0	100.0	100.0	100.0
		(0.57 to 100)	(-5.41 to 100)	(90.82 to 100)	(10.58 to 100)	(100 to 100)	(100 to 100)	(100 to 100)	(100 to 100)
ASMQ+PQ	4	99.95	99.38	100.0	100.0	100.0	100.0	100.0	100.0
		(99.82 to 100)	(97.50 to 100)	(100–100)	(100–100)	(100–100)	(100–100)	(100–100)	(100–100)

Inhibition of intensity and prevalence is the mean percentage reduction (relative to baseline) in number of oocysts per mosquitoes and mosquito infection rate at the given timepoints; positive values denote reduction in percentage of mosquitoes infected and number of oocysts per mosquitoes, negative values denote increases. Full details of mosquito feeding assay outcomes are shown in supplementary data S2.

At 24 h after the beginning of treatment (1 day), none of the participants in the CQ+PQ and ASMQ+PQ groups infected any mosquitoes (Fig 2A and 2C), while three of the four participants in the CQ+TQ group infected mosquitoes (Fig 2B and S2A Table). However, the mean of prevalence of inhibition of CQ+TQ was 66.84% (range 10.58 to 100) and intensity inhibition was 96.10% (range 90.82 to 100) (Fig 3B and 3F and Tables 2 and S1). After 48 h from the start of treatments, all the treatments showed 100% prevalence for inhibition (Fig 3C and 3D and Table 2).

**Fig 4:**
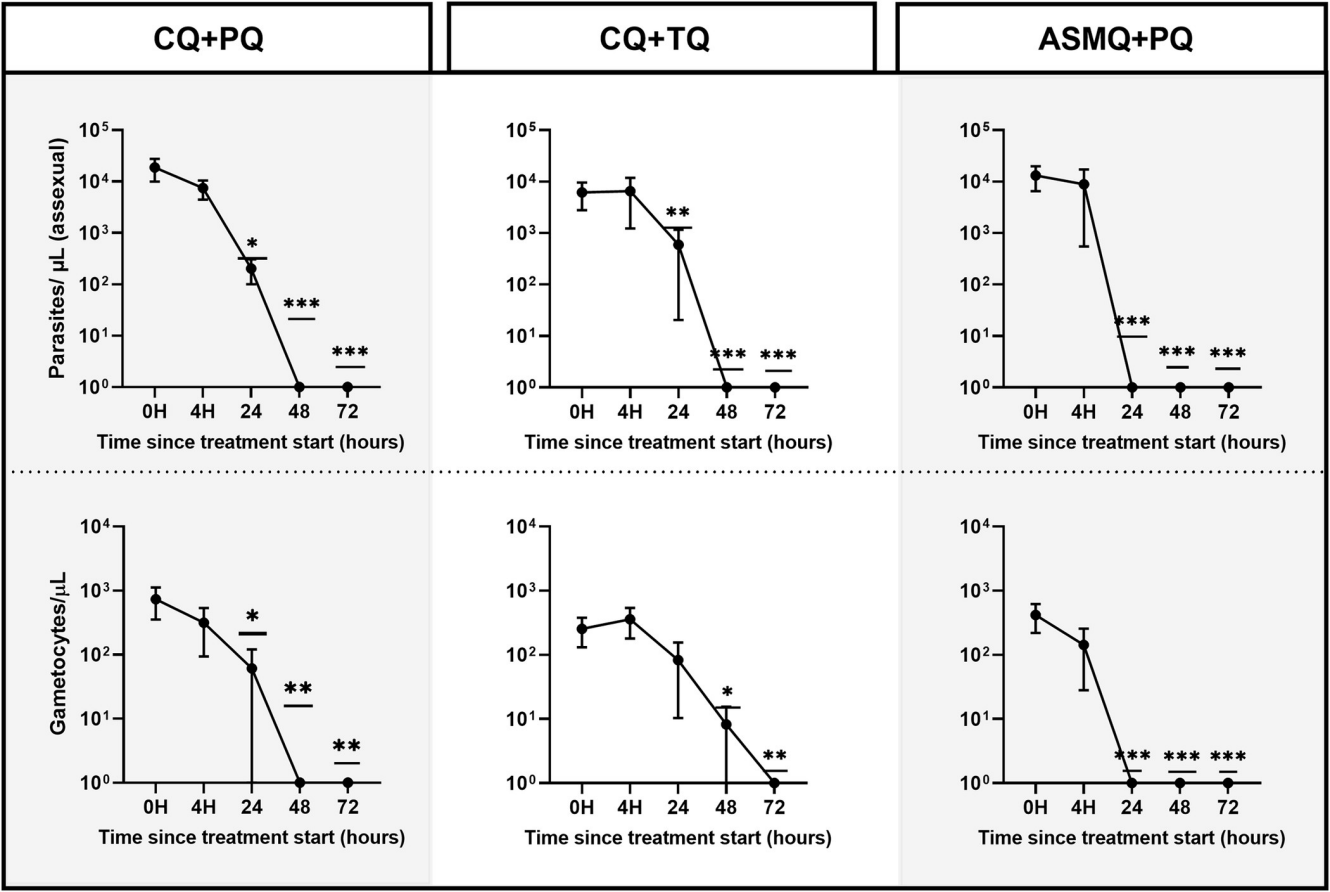
Parasitemia and gametocyte density obtained in each treatment for the timepoints. Black circles indicate the mean of parasitemia and gametocytemia of all patients. Error bars indicate standard error of mean. Asterisks indicate the statistical difference against the control group (0 h). Only significant differences are shown, * p<0.05; ** p<0.01; *** p<0.001.

Regarding parasitemia density, 91.7% of patients had gametocytemia on admission and a median of 5,445 asexual parasites/μL. Individuals who were gametocytemia negative at 0 h (before beginning of treatment) infected mosquitoes (S3 Table). In general, gametocytemia densities declined over time in all the treatment groups, though much more rapidly in those who received ASMQ+PQ (Fig 4C). The same profile was registered in asexual parasite densities, though much more rapidly in those who received CQ+TQ and ASMQ+PQ (Fig 4B and 4C). Although the gametocyte stage was identified at 24 h and 48 h after beginning the CQ+PQ and CQ+TQ treatment (Fig 4D and 4E), mosquitoes were infected only at timepoint 24 h of CQ+TQ treatment (Fig 2E and 2H). Additionally, although gametocyte density was registered for those whose received ASMQ+PQ, there were no significant differences at the 0 h and 4 h timepoints (Fig 4F). Only one patient was infectious to mosquitoes (1/4) at 4 h (Fig 2F and 2I and S2 Table).

## Discussion

In this study, we assessed the efficacy of the transmission blocking by using a DMFA with the blood from patients who received first-line treatment with CQ+PQ, patients who participated in TQ implementation studies and received CQ+TQ, and patients who returned to the clinic with vivax malaria from days 14 to 60 after first-line treatment and received ASMQ+PQ. Our data showed that only one of four patients who were treated with ASMQ+PQ were able to infect mosquitoes for up to 4 h. While three of four patients treated with CQ+PQ or CQ+TQ were able to infect mosquitoes for up to 4 h. However, only patients treated with CQ+TQ remained infective to mosquitoes at 24 h after initiation of treatment. Artemisinin compounds have already been described as faster in clearing asexual *P*. *vivax* parasites and blocking human-to-mosquito transmission when compared with chloroquine [16,34–37]. Artemisinin-based combination therapy (ACT) has been adopted in areas where the efficacy of chloroquine is decreasing. For *P*. *falciparum* treatment, artemisinin derivates prevent gametocyte development, while primaquine is the only drug known to act on mature gametocytes [1]. Primaquine has been used for radical cure of *P*. *vivax* [38] and also kills the asexual blood stages of *P*. *vivax* in therapeutic doses [37], as well as neutralizes the infectivity of mature *P*. *vivax* gametocytes to mosquitoes [39]. Klein et al. [40] showed that, when combined, chloroquine and primaquine required at least 5 h to affect transmissibility, and these data agree with the data reported in the present study. However, the elimination of gametocytes detected by microscopy, even as asexual parasites, was registered only at 72 h after initiation of the treatment. A possible explanation is that the gametocytes found at the timepoints 24 h and 48 h were either inviable or insufficient to infect the *An*. *darlingi* mosquitoes.

The transmission-blocking activity of antimalarial drugs is an important determinant of their impact on public health. Early diagnosis and treatment of malaria are essential for malaria control, mainly for *P*. *vivax* infection due to the rapid appearance of gametocytes in the peripheral blood. Unlike *P*. *falciparum*, *P*. *vivax* gametocytes tend to be already present when patient seek diagnosis and treatment [16,41,42]. Despite the short life of the *P*. *vivax* gametocyte (a maximum of 3 days) [43], the gametocytes must be eliminated as soon as possible to the limit risk of transmission.

Recently in Brazil, tafenoquine, a new drug for the radical cure of *P*. *vivax*, was approved and has been implemented in two cities of Brazil (Manaus and Porto Velho) [12,21,28]. The treatment with chloroquine plus tafenoquine offers an alternative option for preventing relapses and reducing transmission of *P*. *vivax*. Tafenoquine exerts an extraordinarily broad spectrum of activity including against the exo-erythrocytic liver and erythrocytic asexual forms, sexual stages and sporogonic development in the mosquito host [44–49]. In our study, we assessed for the first time the action of tafenoquine in a transmission-blocking assay, using a DMFA with *An*. *darlingi*. We found that three of four treated patients maintained the infection for up to 24 h, with the infection intensity ranging from 1 to 58 oocysts per midgut. Gametocytes were detected for up to 48 h and asexual parasites for up to 24 h. Tafenoquine is a slowly eliminated 8-aminoquinoline (terminal elimination half-life of approximately 2 weeks) that contrasts with the very rapid elimination of primaquine (half-life of 4 h) [50]. The long half-life could be an advantage as a single-dose treatment for the radical cure of *P*. *vivax* as it may prevent relapses of *P*. *vivax* malaria [21]. Ponsa et al. [49] also suggested that the long half-life of tafenoquine increased the probability that the sporogonic-stage parasites might be exposed to the drug, which also affects *P*. *vivax* transmission.

It was demonstrated herein that ASMQ+PQ acts faster than the two other treatments, however, this treatment is available for patients who presented recurrence, which means that these patients had already received the first-line treatment up to 60 days previously. The first-line treatment for *P*. *vivax* in Brazil (CQ+PQ) showed transmission blocking of < 24 h, preventing the risk for transmission of parasites to the mosquito vector the night after taking the first dose. Therefore, protection of patients from bites of malaria vectors for at least 24 h after the initial dose should be used to interrupt the man-mosquito cycle malaria.

Data from CQ+TQ treatment should be continue investigated, considering that it is a new drug and because it is included as an option for *P*. *vivax* treatment in Brazil. A few studies have systematically characterized the gametocytocidal or sporontocidal properties of new antimalarial drugs [19,20,49]. The continued malaria transmission at 24 h is maybe due to the metabolism concentration of tafenoquine, since it does not reach a high level as quickly as primaquine; however, due the long half-life of tafenoquine, it probably maintains the concentration in the metabolism for more time. Therefore, in the future, it will be interesting to add in our study further investigation about the metabolism concentration of these two antimalarials and include more patients. This study is limited by its small sample size, which means that the true impact of each drug at different time points after treatment may not be the same at the population level. Notwithstanding, our study provides a platform for the evaluation of new antimalarials, such as tafenoquine, and other gametocytocidal compounds that may impact the epidemiology of malaria.

## Supporting information

S1 Table Individual data from Intensity and prevalence inhibition in mosquito infection for individuals who were infectious to mosquitoes after beginning of treatment. (XLSB)

S2 Table Infectivity to mosquitoes. (XLSB)

S3 Table Data from DMFA using patients treated with Cloroquine + Primaquine (CQ + PQ), Cloroquine + Tafenoquine (CQ + TQ) and Artesunate and Mefloquine + Primaquine (ASMQ + PQ). (XLSB)
